# Inhalers, inhalers everywhere, but not a proper puff 

**DOI:** 10.7196/AJTCCM.2019.v25i1.004

**Published:** 2019-04-12

**Authors:** Richard van Zyl-Smit, Tarig Ahmed

**Affiliations:** 1 Division of Pulmonology and UCT Lung Institute, Department of Medicine, University of Cape Town and Groote Schuur Hospital, Cape Town, South Africa; 2 Division of Pulmonology, Department of Medicine, University of Cape Town and Groote Schuur Hospital, Cape Town, South Africa

## Editorial

'Adherence’, ‘compliance’, ‘taking your medication correctly’. Whatever you wish to call it, adherence (the preferred term) is critical for managing an underlying condition effectively. Lack of adherence probably contributes considerably to the drug resistance issues seen in tuberculosis, poor glycaemic control and vascular complications in diabetes, and stroke and heart attacks in hypertension.

This is true in the case of asthma as well. In 2014, South Africa (SA) reported the highest age-standardised asthma mortality rate globally, with more than 280 deaths per million population.^[1]^ Fortunately, we have since moved from the top spot: the 2018 Global Asthma Report now places us third, with a mortality rate of just over 100 per million population.^[2]^ There are several potential reasons for this high rate: from lack of diagnosis, poor education on the disease, deficiencies in provision of regular medication (currently a major problem due to stockouts and random switching of inhalers) to simple non-adherence.

Adherence – or the lack thereof – is a subject on its own, with many studies showing that clinicians vastly overestimate patients’ adherence. In airway diseases, there is an additional element to adherence, not present in ‘tablet-based’ therapy. If a hypertensive patient confirms having ‘taken their medication’, the clinician can be fairly sure that, provided the patient swallowed, the drug was delivered to the body. This is not true in the case of asthmatic patients: they can be 100% adherent and use their inhalers twice daily every day, but if their technique is poor, no medication may be delivered to the lung. Perfect adherence, but no clinical effect.

Social stigma and embarrassment can contribute to poor adherence with asthma inhalers. Gupta *et al*.^[3]^ studied the effect of social beliefs and perceptions on inhaler use in India and found that 84.2% of respondents who were not asthmatic considered inhalers a social stigma. There was no gender bias, but older patients seemed to be stigmatised more for using an inhaler. In another example, patients in Sudan prefer to regard their condition as an allergy rather than asthma, owing to incorrect beliefs about inhalers and their association with asthma. In SA, there is a belief that inhaled medication ‘makes the heart weak’. Unlike in the case of hypertension, but similar to that for type I diabetes, the potential requirement for the use of medication in public impacts on adherence.

In this issue of AJTCCM, Ramkillawan *et al*.^[4]^ report on patient inhaler technique in a small study from Bloemfontein. In a convenience sample of 100 patients, only 13 demonstrated completely correct technique. This is slightly uncomplimentary towards the patients, as a third only made a single error. However, what is of greater concern is that a third made more than three errors, with five patients not taking the cap off the inhaler!

Apart from not taking the cap off, which is considered an unequivocal ‘critical’ error, the other steps vary in their importance for appropriate drug delivery, and it has not been described well in the literature as to which are truly critical and which are not.^[5]^ The observer’s assessment of whether each step was correct or not – especially with regard to inhalation and breath holding – is also at play. Is it a more serious error to not breathe out fully, or to not hold your breath for 10 seconds? Similarly, which is more critical: breathing in too fast or not shaking the inhaler first? Each error or combination of errors will impact differently on clinical outcomes, but are very difficult to study in clinical practice.

Where does this leave us? Data from a 2016 systematic review by Sanchis *et al*.^[6]^ suggest that inhaler technique is universally poor and has not substantially improved since 1975. Can South Africans do better? We have the revolutionary Red Cross Hospital 500 mL plastic bottle spacer.^[7]^ In Africa, adherence rates for antiretroviral therapy are higher than in the USA and Europe, especially among adolescents.^[8]^

We need innovative African solutions to teaching and training of inhaler technique. We need to change perceptions and reduce stigma about asthma and chronic obstructive pulmonary disease. We need to promote the use of spacers to assist with drug delivery. We need to ensure that our patients consistently get the correctly prescribed medication from the pharmacy and use it optimally.

If SA has the ability to invent the Kreepy Krauly, the dolos and computed axial tomography technology, and could give the world the first heart transplant, could we not innovate and improve on inhaler technique and adherence too?
